# Taste loss as the sole presenting symptom in Chinese patient with facial onset sensory and motor neuronopathy

**DOI:** 10.1111/cns.13755

**Published:** 2021-11-02

**Authors:** Lu‐Xi Chen, Gong‐Lu Liu, Hao Yu, Zhi‐Ying Wu, Hong‐Fu Li

**Affiliations:** ^1^ Department of Neurology and Research Center of Neurology in Second Affiliated Hospital, and Key Laboratory of Medical Neurobiology of Zhejiang Province Zhejiang University School of Medicine Hangzhou China

**Keywords:** amyotrophic lateral sclerosis, Chinese, facial onset sensory and motor neuronopathy, taste loss

## CONFLICTS OF INTEREST

The authors declare that there is no conflict of interest.

## CONSENT TO PARTICIPATE

The patient provided written consent for participation.

## CONSENT FOR PUBLICATION

The patient provided written consent for disclosure of medical information and images.

Dear Editors,

First reported by Vucic in 2006,^1^ facial onset sensory and motor neuronopathy (FOSMN) syndrome is a rare progressive neurological disease, with a total of 100 patients reported in the literatures worldwide.^2^ However, no cases have been reported from Chinese centers. The age of onset is usually during the fourth to seventh decade with only one case of childhood onset reported.^3^ There is a male predominance, with nearly two‐thirds of cases seen in men, and the disease duration ranges from 1.2 to 46 years with an average duration of 8.2 years.^2^ Patients of FOSMN initially present with sensory deficits of the trigeminal nerve distribution, followed by rostral‐caudal spreading to the scalp, neck, upper trunk, and upper limbs.^1^ Later in the course of the disease, bulbar dysfunction (dysarthria and dysphagia) occurs. Then, lower motor neuron (LMN) features present, including muscle weakness, atrophy, and fasciculation. Generally, only the lower motor neuron system is involved, while the upper motor neuron involvement is rarely seen.^4^ Herein, we described a Chinese patient with FOSMN who initially presented with taste disturbance.

## 1 CASE PRESENTATION

**FIGURE 1:**
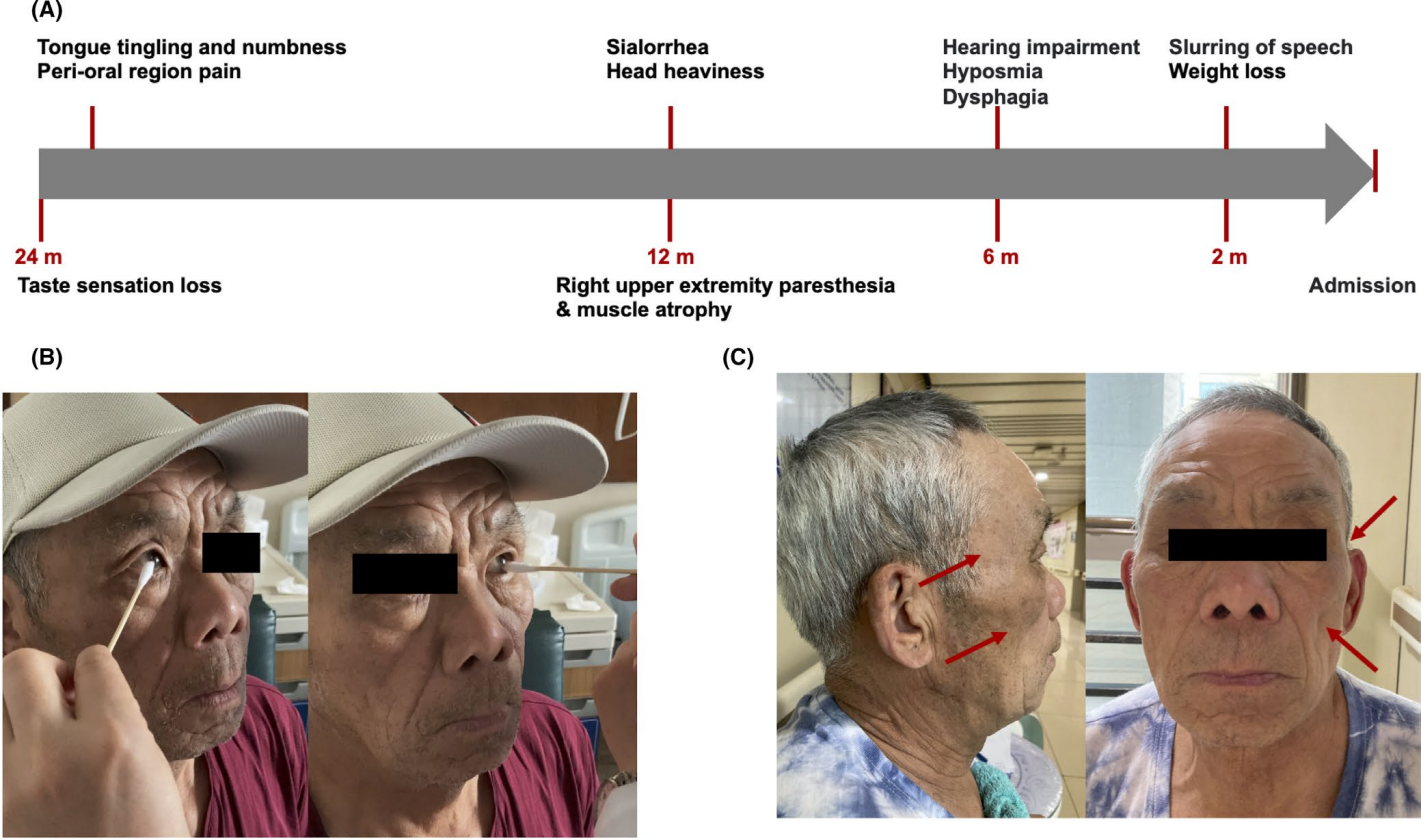
Clinical history and cranial nerves examinations of FOSMN patient. (A) The onset timeline of symptoms in the FOSMN patient. (B) Absent bilateral corneal reflexes. (C) Atrophy of the bilateral temporal and masseter muscles

A 72‐year‐old Chinese man presented to our neurology clinic complaining of progressive loss of taste over 2 years. He was unable to differentiate spicy, salty, or sweet tastes. One month after the onset, he noticed constant tingling and numbness at the tip of his tongue and pain in the peri‐oral region, which disrupted his sleep. He was prescribed gabapentin, and the pain was relieved. One year later, he exhibited paresthesia in his right upper limb, followed by muscle atrophy. At the same time, he developed sialorrhea and headache. One and a half years after initial presentation, he developed dysphagia, hearing impairment, and hyposmia. In the latest 2 months, his dysphagia deteriorated, and he developed dysarthria with losing 5 kilograms of weight. The total timeline of symptom onset is shown in Figure 1A. He sought his medical care in the local hospital and was diagnosed with “progressive bulbar palsy.” His family history was unremarkable.

Cranial nerves examinations revealed absent corneal reflexes (Figure 1B), reduced gag reflex, and loss of taste sensation in the entire tongue. There was no tongue atrophy or fasciculation. Light touch sensation and pinprick sensation were diminished in the peri‐oral distribution and right limb. Vibration and proprioception sensations were normal. Medical Research Council (MRC) grade of neck flexion was 4‐. Muscle atrophy was seen in the bilateral temporal and masseter muscles (Figure 1C) and in the right upper limb (deltoid muscle, biceps muscle, and subscapularis muscle). Strength was normal in all limbs. Deep tendon reflexes were normal in the upper limbs, but brisk in lower limbs. Bilateral palmomental reflexes were positive. There was no extrapyramidal dysfunction, cerebellar ataxia, nor dysautonomia. Systemic examination was unremarkable. A Mini‐Mental State Examination (MMSE) score was 30 out of 30. The Revised Amyotrophic Lateral Sclerosis Functional Rating Scale (ALSFRS‐R) score was 43.

**FIGURE 2:**
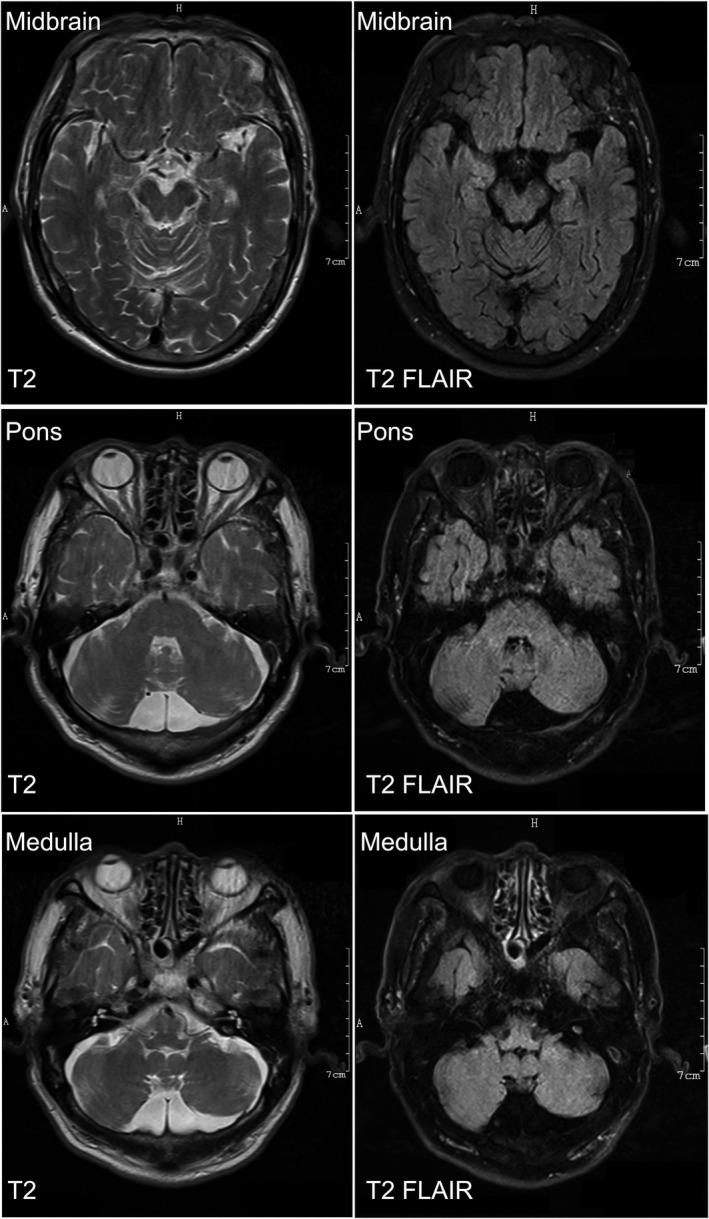
No abnormality found in brainstem (midbrain, pons, and medulla) on MR axial T2 and T2‐weighted‐Fluid‐Attenuated Inversion Recovery (T2‐FLAIR)

Results of complete blood count, creatine kinase, electrolytes, total cholesterol and triglyceride, liver function, renal functions, blood sugar, HbA1c, and C‐reactive protein were normal. Nerve conduction study showed sensory nerve action potentials (SNAP) decreased in the right median nerve, ulnar nerve, and both superficial peroneal nerves. In needle electromyography, extensively chronic neurogenic changes were apparent in muscles of the bulbar, cervical, thoracic, and lumbosacral regions. A pulmonary function test showed that his forced vital capacity (FVC) was 59.3%. Magnetic resonance imaging scans of the brainstem showed no abnormality (Figure 2). Genetic testing of the whole exome sequence was negative. Abnormal nucleotides repeat expansions of the AR or C9ORF72 genes were negative.

## 2 DISCUSSION

There have been two cases of FOSMN reported with initially taste disturbances in patients of Caucasian and Japanese descent respectively. The Caucasian patient was a 42‐year‐old man with taste sensation loss, paresthesia, and numbness in the peri‐oral area. However, his numbness did not progress to the head and neck until 5 years later.^1^ The Japanese patient was a 49‐year‐old man who presented solely with taste disturbance, and other manifestations developed more than 4 years later.^5^ Different from these two cases, our Chinese patient with FOSMN presented with only taste loss initially, but developed tongue numbness and peri‐oral pain 1 month later. His other symptoms manifested about 1 year later, suggesting more rapid progression in our patient. LMN symptoms are common in patients with FOSMN, while only a few cases also have UMN symptoms.^6^ Other reports indicate that the development of UMN signs is associated with more rapid disease progression.^7^ Our case also corroborates this association, with examination finding of UMN signs and a more rapid disease course.

Our case met the diagnostic criteria of amyotrophic lateral sclerosis (ALS) according to the revised El Escorial criteria, which may be the reason for initial misdiagnosis. Our case showed no abnormality in magnetic resonance imaging scans of the brainstem. However, recent studies detected neurochemical or glucose‐metabolism abnormalities in brain stem and cortex of ALS animals using magnetic resonance spectroscopy, suggesting that the above abnormalities may contribute to early differential diagnosis between patients with ALS and FOSMN.^8^, ^9^ It remains controversial whether FOSMN is a neurodegenerative disease, an inflammatory disease, or a mix of pathophysiological mechanisms. Minimal response to immunomodulation therapy with refractory progression suggests that FOSMN probably falls into the neurodegenerative spectrum. In addition, FOSMN patients can carry mutations in *SOD1*, *TARDBP*, and *SQSTM1*,^2^ indicating a potential overlap between FOSMN and ALS. In addition, TDP43 pathology, which is associated with ALS, was detected in the brainstem and spinal cord of FOSMN cases at autopsy.^10^ TDP43‐positive inclusions were seen in both motor (anterior horn cells) and sensory (dorsal root ganglia) neurons in FOSMN, while sensory neurons were spared in ALS.^10^ Sensory functions are commonly spared in ALS patients. However, a minority of patients with ALS do have sensory disturbances, including taste change.^11^ Nevertheless, there have been no reported cases of ALS with taste disorder as the initial manifestation.^5^

Steroids, immune modulation, and intravenous immunoglobulin have not been effective for most patients with FOSMN. Only three reported patients have benefited, with only one having long‐term effect and the other two patients having only transient improvement.^2^, ^12^ Considering the controversial therapeutic effects and the definite side effects of steroids, we did not use immunomodulatory therapies in our patients. It has been reported that the combined use of the ALS‐targeted drug riluzole and the anti‐inflammatory drug celecoxib presented neuroprotection in FUS‐tg mice, which may imply the potential benefits in FOSMN.^13^

To our knowledge, this is the first report of a Chinese patient with FOSMN presenting with taste deficits as the initial symptom, with subsequently presbycusis and hyposmia. More importantly, patients with LMN and UMN signs may mimic ALS at first sight, though the onset of taste disturbance heralds the diagnosis of FOSMN.

## Data Availability

The original contributions presented in the study are included in the article, further inquiries can be directed to the corresponding authors.
